# Epigenetic Mechanisms, Assessment and Therapeutics of Epidermal Stem Cells in Skin Aging

**DOI:** 10.1155/sci/7575250

**Published:** 2025-12-18

**Authors:** Jiayu Yang, Mohan Xu, Yiheng Duan, Yuhang Yuan, Jiaming Zhang, Wenqing Jiang

**Affiliations:** ^1^ Department of Plastic and Cosmetic Surgery, Nanfang Hospital, Southern Medical University, 1838 Guangzhou North Road, Guangzhou, Guangdong, 510515, China, fimmu.com

**Keywords:** cellular senescence, epidermal stem cells, epigenetic clock, epigenetic regulation, skin aging

## Abstract

Skin aging is a multifaceted biological process driven by genetic and environmental factors, in which epidermal stem cells (EpSCs) decrease in number and decline in function. Emerging evidence indicates that epigenetic modifications play a crucial regulatory role in the aging process. Therefore, elucidating the epigenetic mechanisms in aging will provide novel avenues for developing strategies to delay aging. In this review, we explore the epigenetic mechanisms regulating EpSCs function, namely DNA methylation (DNAm), histone modifications, noncoding RNA, and their dysregulation and the resulting series of manifestations during aging. Furthermore, we introduce epigenetic clocks such as Horvath’s and the skin‐specific VisAgeX to quantify these age‐related changes, which provide precise biomarkers of biological age, enabling the assessment of both aging progression and therapeutic outcomes. Finally, we summarize emerging interventions targeting these epigenetic disruptions. Advancing these epigenetic modulations holds significant potential for cutaneous antiaging and fostering innovative dermatological treatments.

## 1. Introduction

Skin aging is a complex biological phenomenon resulting from the interplay of intrinsic factors and extrinsic influences [1]. This process significantly impairs the regenerative capacity of epidermal stem cells (EpSCs), which are pivotal for maintaining skin homeostasis [1]. The function of EpSCs is tightly regulated by epigenetic mechanisms, including DNA methylation (DNAm), histone modifications, and noncoding RNAs, all of which precisely orchestrate gene expression [2–4]. Importantly, these epigenetic controls are increasingly recognized as key contributors to the age‐related decline in EpSCs efficacy, thereby diminishing skin resilience [5]. The interplay between EpSCs and epigenetic regulation offers critical insights into skin aging mechanisms and potential interventions. Epigenetic clocks, such as Horvath’s clock, utilize DNAm patterns to estimate biological age [6, 7]. These clocks not only provide a precise measure of cellular aging but also serve as powerful tools to uncover age‐associated epigenetic shifts in skin cells, like VisAgeX focusing on skin‐specific epigenetic aging patterns [8–10].

In this review, we provide a systematic analysis of the changes in EpSCs within the epidermis during skin aging and explore the underlying epigenetic mechanisms that drive their aging. Additionally, we summarized the role of epigenetic clocks in quantifying cellular aging and evaluated emerging therapeutic strategies aimed at delaying skin aging. By synthesizing recent research findings, this study seeks to elucidate the potential of epigenetic interventions targeting EpSCs to counteract age‐related skin deterioration, thereby advancing both therapeutic and esthetic applications.

## 2. Changes Associated With EpSCs in the Epidermis During Skin Aging

EpSCs serve as the cornerstone of epidermal homeostasis, driving continuous renewal of keratinocytes to sustain a robust skin barrier. As skin ages, its capacity for proliferation and differentiation wanes, initiating a cascade of changes that define the aging phenotype.

### 2.1. Histological and Functional Decline of the Aging Epidermis

Aging precipitates a significant thinning of the epidermis, documenting a 20%–30% reduction in thickness by age 70 [11]. This attenuation stems from a diminished EpSCs‐driven renewal of keratinocytes, observable in hematoxylin and eosin (HE)‐stained sections as fewer stratum corneum layers and a sparser basal cell population [12, 13]. The functional fallout is profound, as the skin’s barrier weakens, leading to increased transepidermal water loss, persistent dryness (xerosis), and greater susceptibility to infections [14]. Concurrently, the dermal‐epidermal junction (DEJ) loses its characteristic undulations, flattening due to impaired basal cell turnover. This structural change, represented as a straighter DEJ contour, undermines the mechanical stability and nutrient exchange between the epidermis and dermis [15]. These deficits manifest most strikingly in wound healing. Aged mice with sluggish EpSCs mobilization exhibited closure rates that were reduced by up to 50% compared with their younger counterparts. Such delays elevate the risk of chronic wounds in the elderly [16, 17]. And external factors like ultraviolet (UV) exposure make this worse. UV accelerates EpSCs decline and ushers in photoaging presented as pronounced thinning and loss of elasticity [18, 19].

The senescence of EpSCs underpins the macroscopic changes observed in aging skin. Thus, it is vital to understand the changes in EpSCs at the cellular level. Within the basal layer, senescent EpSCs exhibit nuclear alterations, including increased chromatin condensation and nucleolar fragmentation, which appear as darker, more clumped nuclear staining [20]. These changes signal DNA damage accumulation and a shift toward a senescent state, impairing the regenerative potential of the cells. Additionally, the reduction in organelle content, particularly mitochondria and endoplasmic reticulum (ER), is inferred from paler cytoplasmic staining, suggesting diminished metabolic activity [21]. In contrast to the uniform, densely packed basal layer of youthful skin, aged skin reveals larger, and darker EpSCs nuclei along with reduced cell density [22].

### 2.2. Cellular Reprograming of EpSCs: Adhesion, Proliferation, and Migration Loss

EpSCs anchor to the basement membrane via integrins, but aging reduces both the expression and function of these receptors. This decline lays the groundwork for the DEJ collapse [23, 24]. Diminished *α*3*β*1 integrin levels compromise stable keratinocyte adhesion to the laminin‐rich matrix, preventing the formation of an ordered basal layer [25]. Concurrent defects in *α*6*β*4 integrin weaken hemidesmosomal attachments to Laminin‐5 and suppress Rac1 activation [26]. In the absence of these critical mechanical cues, coordinated collective migration is disrupted, resulting in erratic cell dispersal and impaired wound closure [26, 27]. Simultaneously, chronic inflammation in aged EpSCs activates the Jak‐STAT signaling pathway. This activation, in turn, drives the upregulation of EMT factors such as Snail, Zeb1, and Twist‐1, which should heighten cell motility. This potential is, however, undermined by flawed integrins that prevent the cells from securing the firm adhesion needed for effective migration [28–30].

This incoordination is exacerbated by imbalances in intracellular signaling. In the aged, pro‐inflammatory microenvironment, Protein Kinase C (PKC) exhibits atypical activation [31], and contrary to its typical role, inhibits growth factor (e.g. EGF)‐induced keratinocyte migration [32]. Furthermore, the damage‐associated keratins KRT6 and KRT16, while critical for mechanical strength, adopt a dysregulated mode of action under chronic inflammation that disrupts directional sensing [33, 34]. As a result, EpSCs are stalled in a hypermetabolic, inflammatory state that lacks the coordination required for effective tissue repair [35, 36].

### 2.3. Molecular Drivers and Pathways Associated With EpSCs During Skin Aging

There are changes in some factors and pathways associated with EpSCs during skin aging. Elevated levels of cytokines, such as IL‐6, regulate EpSCs behavior through signaling pathways including Jak‐STAT, Hippo and Notch potentially diminishing their regenerative capacity [13, 37–39]. The inhibition of Jak‐STAT signaling exacerbates regenerative decline, mirroring the delayed wound healing observed in aged murine skin [16, 40]. Moreover, the secretory activities of other cell types can modulate the extracellular matrix (ECM) to influence EpSCs in aged skin. For instance, fibroblasts produce migrasomes, a process mediated by Tetraspanin 4 (TSPAN4) that becomes impaired during skin aging [41]. Consequently, young fibroblast‐derived migrasomes have been demonstrated to enhance the migration of senescent HaCaT cells, reduce reactive oxygen species (ROS) levels and downregulate pro‐inflammatory factors including IL‐1*β*, IL‐6, and MMP‐14, thereby facilitating epidermal repair [42].

**Figure 1:**
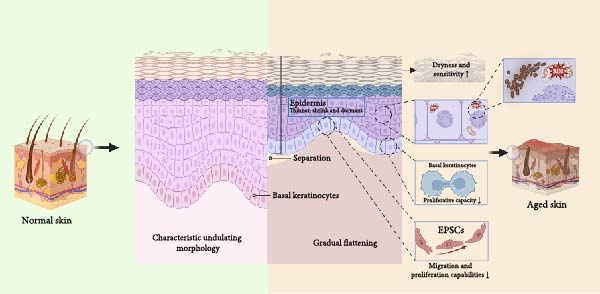
Macroscopic manifestations of epidermal cells associated with EpSCs in skin aging. The aged epidermis shows impaired barrier function, thinner stratum corneum, atrophic basal membrane zone, and flattened dermo‐epidermal junction, which are all related with the declining of EpSCs function. The self‐renewal capacity of aged EpSCs decreases, and thus the aged keratinocytes perform worse in adhesion, migration, and proliferation, which causes an increasing dermo‐epidermal separation, further leading to a poor supply of nutrients for epidermis. Microscopically, fragmented nucleoli, swollen mitochondria, and fragmented endoplasmic reticulum are shown in the aged EpSCs.

In human studies, the Hippo and Notch signaling pathways, which are crucial for maintaining skin integrity, become dysregulated with age [39, 43]. Aging‐associated environmental stressors such as UV radiation and oxidative stress, may lead to mutations in the LAMB3 gene, which encodes the *β* 3 chain. *β* 3 chain is a crucial component of Laminin‐332 [44, 45]. Such mutations result in a defective Laminin‐332 protein in the basement membrane, severely compromising the integrity of the ECM and the mechanosensing function of EpSCs. This loss of ECM integrity serves as a direct signal for the dysregulation of the downstream Hippo pathway [46–48]. The activity of key Hippo effectors, yes‐associated protein (YAP) and its paralog TAZ decreases, disrupting the balance between stem cell proliferation and differentiation [38, 49]. This is exemplified in junctional epidermolysis bullosa (JEB), where YAP inactivation leads to EpSCs depletion [50]. In addition, declining Notch activity disrupts the conversion of EpSCs into mature keratinocytes [39]. These collective deficits in self‐renewal and differentiation contribute to the characteristic thinning and fragility of aged skin [51, 52]. These molecular dynamics underscore the complex interplay between cytokine signaling, ECM components, and EpSCs senescence in driving the aging phenotype (Figure 1).

## 3. Epigenetic Mechanisms of EpSCs in Skin Aging

### 3.1. Epigenetic Dysregulation and Gene Level Changes

There is ample evidence that epigenetic mechanisms contribute significantly to skin aging because of their sensitivity to lifestyle and environmental factor [53, 54]. Epigenetic mechanisms include histone modifications DNAm and changes in chromatin structure. Endogenous and exogenous factors collectively drive diverse epigenetic manifestations of senescent phenotypes at the cellular level during skin aging.

During senescence, the level of histone H4 on arginine 3 (H4R3me2as) demethylation diminished. Because protein arginine methyltransferase 1 (PRMT1)‐mediated asymmetric demethylation of H4R3me2as preserves the stability of H4, the reduction of H4R3me2as level in turn leads to the strengthened interaction between proteasome activator PA200 and histone H4, which ultimately catalyzes the polyubiquitin‐independent degradation of histone H4 [55]. Histones are involved in transcriptional regulation, DNA repair, DNA replication, and chromosome stability, and are a core component of nucleosomes. Thus, posttranslational modifications of histones and alterations in DNAm result in the widespread loss of heterochromatin and focal gains of heterochromatin during skin senescence [56, 57].

Meanwhile, DNAm is catalyzed by a conserved family of enzymes known as DNA methyltransferases (DNMTs), which cooperatively establish and maintain DNAm patterns during embryonic development and tissue homeostasis [58]. Among these, DNMT1 plays a pivotal role in preserving methylation patterns during cellular replication by copying methylation marks from the parental (methylated) DNA strand to the newly synthesized daughter strand [59, 60]. DNMT1 maintains a methylation state by adding methyl radicals to cytosine C5 and the 5‐methylcytosine (5‐mC) DNAm is one of the key mechanisms of epigenetics [61]. Notably, in skin epithelial cells, DNMT1 expression exhibits an age‐dependent decline, suggesting its potential contribution to epigenetic dysregulation in aging skin [62]. Beyond DNMTs, the methylcytosine dioxygenase TET3 also plays a critical role in aging through epigenetic regulation [63]. TET3 mediates active DNA demethylation by oxidizing 5‐mC, establishing and maintaining hypomethylated states at critical genomic regulatory regions [64].

**Figure 2:**
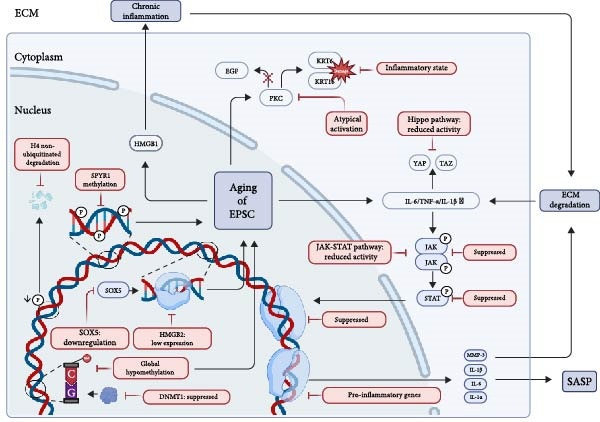
Multiscale epigenetic dysregulation in aging EpSCs. Aged EpSCs initiate a self‐perpetuating aging cycle characterized by epigenetic dysregulation, including DNMT1 suppression‐mediated global hypomethylation, SOX5 downregulation, and non‐ubiquitinated H4 degradation, coupled with HMGB1 translocation. These alterations trigger chronic inflammation, which in turn suppresses Hippo and JAK–STAT signaling and promotes a SASP‐driven ECM degradation feedback loop, collectively arising a cascade decline of epidermal function and even the whole skin.

UV radiation is a major driver of extrinsic aging, which causes epigenetic changes such as hypermethylation of tumor suppressor genes and hypomethylation of oncogenes [65]. Notably, UV exposure triggers global DNA hypomethylation in epidermal keratinocytes, indicative of genome‐wide epigenetic instability [64]. Furthermore, UV radiation modulates epigenetic regulation by altering the expression of key genomic regulators, including DNMT1. In UV‐irradiated human skin, upregulation of DNMT1 may drive TIMP2 promoter hypermethylation, leading to transcriptional silencing of TIMP2 [66]. In addition, poor nutrition, smoking, stress, and lack of sleep exacerbate skin aging by impairing skin repair (Figure 2).

### 3.2. Gene and Transcriptional Level Changes

Through epigenetic modifications such as methylation, phosphorylation, and changes in histones, the gene and transcription level also change accordingly during skin aging.

Concerning the regulation of phosphorylation states, the cellular senescence is marked by the upregulation of cyclin‐dependent kinases (CDK) inhibitors p21^CIPI^ and P16^INK4A^ (also known as CDKN1A and CDKN2A) [67], leading to the persistent hypo‐phosphorylation of RB family proteins, suppression of E2F transcription factor activity, reduced expression of the proliferation marker MKI67 [68], and subsequently cell cycle arrest in the G1 and S phase [69]. Additionally, epigenetic regulation through Sprouty1 (SPRY1) methylation contributes to the natural senescence of skin epidermal cells which are composed of ~95% of keratinocytes [64]. In the immortalized human keratinocyte cell line HaCaT and SPRY1 expression exhibited an age‐dependent increase [62].

In terms of EpSCs, a recent bioinformatics analysis study based on the analysis of the GEO database found the expressions of pro‐inflammatory genes IRF4 and Cxcl12 are upregulated in aging EpSCs, while Peg3 and Cybrd1 are down‐regulated [70]. The changes in these genes may be related to epigenetic regulation. The expression of the cell cycle inhibitor P16^INK4A^ is directly related to chronological aging of the skin in vivo. It has been shown that the number of cell cycle inhibitor P16^INK4A^ protein‐positive cells increases with aging in the epidermis, which may be related to epigenetic changes [71–73]. Notably, senescence‐associated *β*‐galactosidase (SA‐*β*‐gal) activity is significantly upregulated during this process [74]. High mobility group box 1 (HMGB1), a member of the high mobility group protein family known for its role in modulating chromatin architecture and gene expression, is released from the nucleus into the extracellular environment during cellular senescence [75].

In addition, research findings have demonstrated that the level of SOX5 declines in a range of senescent cells and tissues [76]. Moreover, in recent years, it has been found that overexpression of transcription factor SOX5 is sufficient to trigger epigenetic and transcriptional remodeling, leading to the activation of genes such as high mobility group box 2 (HMGB2) and thus alleviating cellular senescence [76].

### 3.3. Altered Cell Structure

Epigenetic changes affect cell morphology through genes expression and transcription. Senescent EpSCs display enhanced degradation of the nuclear fiber layer and nuclear structural abnormalities. These include an enlarged nuclear size, loosened nuclear membranes, chromatin relaxation, decreased levels of Lamin B1 and Lamin‐associated peptide 2 (LAP2), and defects in most of the organelles such as lysosomes, ER, and mitochondria [67]. As a very important heterogeneous organelle for energy metabolism in the human body [77], mitochondria have attracted much attention for their relevance to stem cell senescence. Although inconsistent changes in different models, it has been shown that mitochondrial complex abnormalities lead to elevated intracellular mitochondrial NADH levels and ROS levels [78–81].

### 3.4. Cytokines and Pathways in Metabolism

Under the influence of epigenetics, EpSCs will ultimately affect cytokine release and pathway regulation through the above mechanisms. The aging EpSCs secrete a plethora of chemokines, cytokines, growth factors and proteases including IL‐1*α*, IL‐1*β*, IL‐6, CXCL8, CXCL10, MMP3, MMP9, and MMP1, referred to as the senescence‐associated secretory phenotype (SASP) [82]. IL‐1*α* can elevate the expression levels of TNF‐*α*, IL‐8, IL‐12, MMP‐2, MMP‐9 and IL‐1R1, as well as the ROS level [83]. In addition, aging is also marked by diminished expression of cell adhesion and ECM genes in EpSCs, notably changes in collagen type XVII *α* 1 (COL17A1) [84]. It has been confirmed that COL17A1 is specifically expressed in interfollicular EpSC niches and significantly reduced in naturally aged human skin in vivo [85]. As a binding partner of the aPKC‐PAR complex, COL17, has been implicated in altering cell polarity and aging of the epidermis (Table 1) [88].

**Table 1:** The cytokines related to EpSCs in skin aging.

Marker	Variation	Regulation on EpSCs	Mechanism
Wnt5a [51]	↑Upregulated	Inhibits stemness Loss of polarity	‐Activates noncanonical Wnt/PCP pathway ‐Antagonizes canonical Wnt/*β*‐catenin signaling ‐Reduced EpSCs self‐renewal

IGFBP3 [86]	↑Upregulated	Induces senescence Inhibits proliferation	‐Binds and sequesters (IGF‐1/IGF‐2) ‐Inhibits IGF‐1R signaling pathway ‐Induces EpSCs cell cycle arrest

IL‐1*β* [86]	↑Upregulated	Pro‐inflammatory Induces senescence	‐Activates the NF‐kappaB pathway ‐Induces senescence and a SASP feedback loop

MMP‐3 [87]	↑Upregulated	Loss of anchorage	‐Secreted SASP enzyme ‐Degrades ECM ‐Disrupts basement membrane integrity and EpSCs anchorage

IGFBP‐7 [87]	↓Downregulated	Induces senescence Growth arrest	‐Binds cell surface receptors ‐Activates p53/p21 pathway ‐Induces potent cell cycle arrest

IL‐6 [87]	↑Upregulated	Maintains senescence Inhibits differentiation	‐Activates the JAK/STAT3 signaling pathway ‐Persistent STAT3 activation maintains senescence and inhibits EpSCs differentiation

IL‐1*α* [40]	↑Upregulated	Directly inhibits stem cell clonogenic ability	‐Released as an alarmin ‐Activates the NF‐kappaB pathway ‐Initiates the SASP cascade

*Note:* This table systematically summarizes critical molecular markers related to EpSCs implicated in epidermal skin aging processes, detailing their regulatory pathways, functional impacts on epidermal homeostasis, and interactions with intrinsic and extrinsic aging factors.

Abbreviations: IGFBP‐7, insulin‐like growth factor‐binding protein 7; IGFBP3, insulin‐like growth factor‐binding protein 3; IL‐1*α*, interleukin‐1 alpha; IL‐1*β*, interleukin‐1 beta; IL‐6, interleukin‐6; MMP‐3, matrix metalloproteinase‐3; Wnt5a, wingless‐related integration site 5a.

## 4. Evaluation of Skin Aging With Epigenetic Mechanisms

### 4.1. Current Advances in Epigenetic Clocks for Skin Aging Assessment

Epigenetic mechanisms are recognized as highly promising approaches for assessing skin aging. Among them, DNAm is a well‐studied epigenetic modification with age‐related patterns of change that can be used as a surrogate indicator of biological aging in various tissues. The commonly used DNAm‐based age estimator is called the DNAm clock. In human tissues, epigenetic biomarkers, or biological “clocks” have been developed to reliably track actual age [89]. Epigenetic factors heavily influence aging process. Heritable changes in gene expression do not alter the underlying DNA sequence [6]. This revelation has sparked the introduction of epigenetic clocks as biomarkers of biological aging [10, 54]. The estimate of DNAm age (DNAmAge), also known as the epigenetic clock, is considered one of the most promising indices accurately measuring one’s biological age [90, 91].

### 4.2. Evaluation of Epigenetic Clocks in Skin Aging

Horvath’s clock is one of the most popular epigenetic clocks. It determines biological age in a variety of tissues, including skin, by measuring methylation at the CpG (cytosine–phosphate–guanine) sites in the human genome [7]. It has been supported by data from the field of aging [10, 92]. When it came to predicting skin age, the first generation of Horvath’s clocks applied to pan‐tissues had certain limitations. A novel DNAm‐based biomarker (based on 391 CpGs) was presented using the second‐generation Horvath’s clock. It was created to precisely determine the age of human fibroblasts, keratinocytes, buccal cells, endothelium cells, skin, and blood samples, which were cultivated ex vivo. The dynamic aging of these cells was properly followed by this extremely sensitive age estimator, which also showed that a constant increase in epigenetic age coincides with cell proliferation. Its use on fibroblasts from Hutchinson Gilford Progeria Syndrome patients is an example [93]. The above epigenetic clock uses a wide range of CpG sites, which is insufficiently targeted at skin cells, which may be the reason for its limitations in predicting skin age.

Therefore, to assess skin aging more accurately, incorporating additional skin cell‐specific methylation sites may be desirable. For instance, the EpSCs mentioned in this study could serve as an excellent sample source. A research team has successfully created VisAgeX, a skin‐specific epigenetic aging clock capable of detecting variations in facial visual aging patterns. Their findings show that DNAm profiles from epidermal samples can effectively predict wrinkle severity, perceived facial age, and visual aging progression [6]. These studies comprehensively illustrate how age‐related changes in DNAm might connect environmental exposures to biological skin aging. We compared the advantages and disadvantages of the updated epigenetic clocks and highlighted the significance of the specialized skin epigenetic clock (Table 2).

**Table 2:** The comparison of three important epigenetic clocks.

Epigenetic clock	Horvath clock [7]	Skin and blood clock [93]	VisAgeX clock [6]
Markers	353 CpGs (selected via elastic net regression)	391 CpGs (selected via elastic net regression)	Low‐methylated regions (LMRs)
Marker source^a^	Pan‐tissue (applicable to most tissues except sperm)	Skin, blood, and related cell types (fibroblasts, keratinocytes, endothelial cells, saliva, and buccal cells)	Skin‐specific LMRs (tissue‐specific regulatory regions)
Applicable samples^b^	Multiple tissues (blood, brain, liver, and heart); poorly calibrated in fibroblasts and breast tissue	Fibroblasts, keratinocytes, blood, saliva, buccal cells, neurons, glia, liver, bone, and ex vivo cultured cells	Skin tissue (epidermal samples from volar forearm)
Applications	General biological age estimation across tissues	Monitoring skin aging phenotypes (wrinkles) and antiaging interventions	Assessing visual skin age progression, UV/pollution effects, and personalized aging studies
Age prediction features	Predicts chronological age; measures cumulative epigenetic maintenance; logarithmic ticking rate during development, linear in adulthood	Predicts chronological and biological age; detects subtle age acceleration; more accurate in predicting isolated cells	Directly predicts visual age progression (deviation from chronological age)
Skin‐specific validation (accuracy)	Limited skin specificity: not optimized for skin aging Accuracy: high correlation with chronological age	Skin‐focused: validated on epidermal samples Accuracy: visual facial age, MAE = 6.54 years (visual facial age); *R* = 0.84 (test set)	Skin‐specific and dynamic: directly predicts visual age progression (deviation from chronological age) Accuracy: age progressio, MAE = 4.67–6.17 years (age progression); *R* = 0.48; *p* < 0.001, superior to traditional CpG models

*Note:* This table summarizes the basic information of three epigenetic clocks, comparing their respective advantages and disadvantages based on their characteristics, as well as their strengths and limitations in assessing skin aging. The comparison highlights the significance of the iterative development of epigenetic clocks.

Abbreviation: MAE, mean absolute error.

^a^Source: The origin of epigenetic markers (CpGs or LMRs) used in the epigenetic clock.

^b^Sample: The biological specimens for which the epigenetic clock is validated.

### 4.3. Other Epigenetic Biomarkers of Cellular Aging

Based on the miRNA expression patterns of healthy individuals, a study developed an epigenetic molecular clock using machine‐learning algorithms. The models used 1856 distinct miRNAs to predict age with a mean absolute error of 10.89 years and 80% accuracy in identifying age groups. According to the findings, miRNA‐based epigenetic clocks are noticeable in the outer layers of the skin and correspond similarly to mRNA or DNAm, but they are more stable than mRNA [94].

## 5. The Treatment of Skin Aging Related to EpSCs

### 5.1. Direct Regulation of Epigenetic Targets

We systematically reviewed therapeutic strategies that directly modulate epigenetic mechanisms in EpSCs. Emerging evidence indicates that certain pharmacological agents and bioactive compounds can benefit cutaneous antiaging and facial rejuvenation by targeting molecular mechanisms in EpSCs. Notably, retinoids modulate DNAm patterns to reverse age‐associated epigenetic alterations, demonstrating their capacity to reset epigenetic clocks and promote cutaneous rejuvenation [54]. In addition, some active skincare ingredients such as dihydromyricetin (DHM), curcumin, and genistein, have gained attention for their ability to inhibit DNMT1 [60, 95, 96]. It has been proven experimentally DHM showed robust inhibition of DNMT1 in biochemical assays [60]. And ectoine downregulates DNMTs (DNMT1, DNMT3a and DNMT3L), thereby reducing global DNAm and reactivating tumor‐suppressor genes. This mechanism suggests its potential as an antiaging agent [97].

### 5.2. Indirect Regulation of Epigenetic Targets

In addition to sites that directly regulate epigenetics, we also have systematically summarized treatment strategies aimed at altering downstream pathways of epigenetic regulation.

Apremilast, a small molecular inhibitor of phosphodiesterase 4 (PDE4), protected EpSCs against IL‐1*α*‐induced impairment in capacities of EpSCs, which can offer the opportunity for cutaneous antiaging [83]. Activated Myd88/TRAF6/NF‐*κ*B signaling pathway induced by stimulation with IL‐1*α* was significantly inhibited by the introduction of Apremilast [83]. And biofermented *Aframomum angustifolium* (BAA) extract contained specific organic acids such as lactic, gluconic, succinic acid, and polyphenols. Treating keratinocyte stem cells (KSCs)‐depleted skin equivalents with it exhibited higher mitotic activity in the epidermis basal layer including EpSCs [98].

Injectable skin fillers offer a wider range of options for delaying skin aging. The positive effects of PLLA microspheres which are increasingly favored as degradable and long‐lasting fillers on EpSCs have been shown on rat epidermis and EpSCs [99].

In terms of regulating gene expression, Amniotic membrane (AM) effectively reduces TGF‐induced phosphorylation of Smad2 and Smad3 in keratinocytes, thereby modulating the expression of cell cycle regulators CDK1A (p21) and CDK2B (p15). Given that these cell cycle factors (p21/p15) are crucial mediators of cellular senescence, their altered expression due to the interference of AM with the TGF‐*β* pathway suggests that AM may hold potential for preventing skin aging by delaying cellular senescence and promoting tissue repair [100]. In lipid‐deficient skin under oxidative stress, wound‐edge keratinocytes exhibit elevated p21^CIP^ expression, which impedes wound healing when overexpressed. Conversely, suppression of p21^CIP^ enhances keratinocyte proliferation and barrier repair, suggesting that small‐molecule p21^CIP^ inhibitors—similar to those used in chemotherapy‐resistant kidney carcinoma—could promote tissue regeneration [101]. Additionally, thyroxine (T4) treatment in organ‐cultured human skin downregulates aging‐related biomarkers, including P16^INK4A^ transcription [102], further supporting its potential role in mitigating age‐impaired repair mechanisms.

Triiodothyronine (T3) was discovered to dramatically increase sirt1 transcription in the human epidermis, a factor linked to genomic stability, in organ‐cultured human skin. Although proliferator‐activated receptor‐*γ* 1‐*α* (PGC1*α*) protein expression only demonstrated a nonsignificant rising trend, T3 also raised PGC1*α* mRNA levels. These modifications imply that T3 might affect the epigenetic modulators of mitochondrial activity [102].

## 6. Conclusion and Perspectives

EpSCs play a vital role in skin homeostasis and regeneration, but their quantity and functionality diminish with age, driving skin aging. Epigenetic mechanisms such as DNAm, histone modifications, and noncoding RNAs regulate EpSCs behavior. Aging disrupts these processes, impairing stem cell performance and resulting in visible signs of aging like wrinkles and reduced elasticity. Recent studies highlight promising therapeutic avenues targeting epigenetic changes. Epigenetic clocks, such as Horvath’s, serve as accurate biomarkers to measure the biological age of skin cells. Pharmacological options, including retinoids and DNMT1 inhibitors, show potential in counteracting age‐related epigenetic shifts. Meanwhile, regenerative strategies, such as adipose‐derived stem cell exosomes and iPSC‐derived micro vesicles, enhance skin structure and function and offer antiaging benefits.

Despite these advances, several challenges remain. Precision in epigenetic modulation is crucial, as nonspecific interventions may cause unintended effects. Effective delivery methods are needed to accurately target EpSCs, and clinical trials must confirm safety and efficacy across diverse groups. Future research should pinpoint specific epigenetic targets—like critical methylation sites or histone marks—unique to EpSCs aging for personalized therapies. Innovations in delivery systems, such as nanoparticle carriers, could improve treatment accuracy. Combining stem cell therapies with epigenetic modulators may amplify these results. Longitudinal studies will be key to ensuring long‐term safety and refining protocols.

In conclusion, unraveling the epigenetic control of EpSCs opens exciting possibilities for combating skin aging. Current therapeutic strategies, including biomarkers, pharmacological interventions, and regenerative medicine approaches, demonstrate considerable potential. Critical challenges remain in achieving target specificity, optimizing delivery mechanisms, and establishing robust clinical validation. As this field progresses, it could revolutionize dermatological care, delivering effective, accessible solutions to enhance skin health and resilience.

## Disclosure

All authors read and approved the final version of the work to be published.

## Conflicts of Interest

The authors declare no conflicts of interest.

## Author Contributions

All authors contributed to researching the data for the article and writing the article. Jiayu Yang, Mohan Xu, and Yiheng Duan drafted the main text and tables. Yuhang Yuan and Jiaming Zhang made the figures. Wenqing Jiang supervised the work and provided the comments and additional scientific information. Jiayu Yang, Mohan Xu, and Yiheng Duan also reviewed and revised the text. Jiayu Yang and Mohan Xu contributed equally to this work and should be considered co‐first authors.

## Funding

This work was supported by the National Nature Science Foundation of China (Grant 82202474) and Science and Technology Projects in Guangzhou (Grant 2023A04J2350).

## Data Availability

The data sharing is not applicable to this article as no new data were created or analyzed in this study.

## References

[1] Wong Q. Y. A. and Chew F. T. , Defining Skin Aging and Its Risk Factors: A Systematic Review and Meta-Analysis, Scientific Reports. (2021) 11, no. 1, 10.1038/s41598-021-01573-z, 22075. 34764376 PMC8586245

[2] Bure I. V. , Nemtsova M. V. , and Kuznetsova E. B. , Histone Modifications and Non-Coding RNAs: Mutual Epigenetic Regulation and Role in Pathogenesis, International Journal of Molecular Sciences. (2022) 23, no. 10, 10.3390/ijms23105801, 5801. 35628612 PMC9146199

[3] Wang K. , Liu H. , and Hu Q. , et al.Epigenetic Regulation of Aging: Implications for Interventions of Aging and Diseases, Signal Transduction and Targeted Therapy. (2022) 7, no. 1, 1–22, 10.1038/s41392-022-01211-8. 36336680 PMC9637765

[4] da Silva P. F. L. and Schumacher B. , Principles of the Molecular and Cellular Mechanisms of Aging, Journal of Investigative Dermatology. (2021) 141, no. 4, 951–960, 10.1016/j.jid.2020.11.018. 33518357

[5] Wagner R. N. , Piñón Hofbauer J. , and Wally V. , et al.Epigenetic and Metabolic Regulation of Epidermal Homeostasis, Experimental Dermatology. (2021) 30, no. 8, 1009–1022, 10.1111/exd.14305. 33600038 PMC8359218

[6] Bienkowska A. , Raddatz G. , and Söhle J. , et al.Development of an Epigenetic Clock to Predict Visual Age Progression of Human Skin, Frontiers in Aging. (2024) 4, 10.3389/fragi.2023.1258183, 1258183. 38274286 PMC10809641

[7] Horvath S. , DNA Methylation Age of Human Tissues and Cell Types, Genome Biology. (2013) 14, no. 10, 10.1186/gb-2013-14-10-r115, 2-s2.0-84886111619, R115. 24138928 PMC4015143

[8] Teschendorff A. E. and Horvath S. , Epigenetic Ageing Clocks: Statistical Methods and Emerging Computational Challenges, Nature Reviews Genetics. (2025) 26, no. 5, 350–368, 10.1038/s41576-024-00807-w. 39806006

[9] Bell C. G. , Lowe R. , and Adams P. D. , et al.DNA Methylation Aging Clocks: Challenges and Recommendations, Genome Biology. (2019) 20, no. 1, 10.1186/s13059-019-1824-y, 249. 31767039 PMC6876109

[10] Duan R. , Fu Q. , Sun Y. , and Li Q. , Epigenetic Clock: A Promising Biomarker and Practical Tool in Aging, Ageing Research Reviews. (2022) 81, 10.1016/j.arr.2022.101743, 101743. 36206857

[11] Quan T. , Molecular Insights of Human Skin Epidermal and Dermal Aging, Journal of Dermatological Science. (2023) 112, no. 2, 48–53, 10.1016/j.jdermsci.2023.08.006. 37661473 PMC13155249

[12] Farage M. A. , Miller K. W. , Elsner P. , and Maibach H. I. , Intrinsic and Extrinsic Factors in Skin Ageing: A Review, International Journal of Cosmetic Science. (2008) 30, no. 2, 87–95, 10.1111/j.1468-2494.2007.00415.x, 2-s2.0-40849118347. 18377617

[13] Giangreco A. , Qin M. , Pintar J. E. , and Watt F. M. , Epidermal Stem Cells Are Retained In Vivo Throughout Skin Aging, Aging Cell. (2008) 7, no. 2, 250–259, 10.1111/j.1474-9726.2008.00372.x, 2-s2.0-40549103466. 18221414 PMC2339763

[14] Luebberding S. , Krueger N. , and Kerscher M. , Age-Related Changes in Skin Barrier Function—Quantitative Evaluation of 150 Female Subjects, International Journal of Cosmetic Science. (2013) 35, no. 2, 183–190, 10.1111/ics.12024, 2-s2.0-84874947831. 23113564

[15] Nishimura E. K. , Granter S. R. , and Fisher D. E. , Mechanisms of Hair Graying: Incomplete Melanocyte Stem Cell Maintenance in the Niche, Science. (2005) 307, no. 5710, 720–724, 10.1126/science.1099593, 2-s2.0-13244287677. 15618488

[16] Keyes B. E. , Liu S. , and Asare A. , et al.Impaired Epidermal to Dendritic T Cell Signaling Slows Wound Repair in Aged Skin, Cell. (2016) 167, no. 5, 1323–1338.e14, 10.1016/j.cell.2016.10.052, 2-s2.0-84995769352. 27863246 PMC5364946

[17] Vu R. , Jin S. , and Sun P. , et al.Wound Healing in Aged Skin Exhibits Systems-Level Alterations in Cellular Composition and Cell-Cell Communication, Cell Reports. (2022) 40, no. 5, 10.1016/j.celrep.2022.111155, 111155. 35926463 PMC9901190

[18] Fisher G. J. , Kang S. , and Varani J. , et al.Mechanisms of Photoaging and Chronological Skin Aging, Archives of Dermatology. (2002) 138, no. 11, 1462–1470, 10.1001/archderm.138.11.1462, 2-s2.0-0036845039. 12437452

[19] Kosmadaki M. G. and Gilchrest B. A. , The Role of Telomeres in Skin Aging/Photoaging, Micron. (2004) 35, no. 3, 155–159, 10.1016/j.micron.2003.11.002, 2-s2.0-1242299775. 15036269

[20] di Fagagna F. d’Adda , Reaper P. M. , and Clay-Farrace L. , et al.A DNA Damage Checkpoint Response in Telomere-Initiated Senescence, Nature. (2003) 426, no. 6963, 194–198, 10.1038/nature02118, 2-s2.0-0344441890. 14608368

[21] Dimri G. P. , Lee X. , and Basile G. , et al.A Biomarker That Identifies Senescent Human Cells in Culture and in Aging Skin In Vivo, Proceedings of the National Academy of Sciences. (1995) 92, no. 20, 9363–9367, 10.1073/pnas.92.20.9363, 2-s2.0-0029047362. PMC40985 7568133

[22] Pathak R. U. , Soujanya M. , and Mishra R. K. , Deterioration of Nuclear Morphology and Architecture: A Hallmark of Senescence and Aging, Ageing Research Reviews. (2021) 67, 10.1016/j.arr.2021.101264, 101264. 33540043

[23] Watt F. M. , Role of Integrins in Regulating Epidermal Adhesion, Growth and Differentiation, The EMBO Journal. (2002) 21, no. 15, 3919–3926, 10.1093/emboj/cdf399, 2-s2.0-0036683057. 12145193 PMC126145

[24] Roig-Rosello E. and Rousselle P. , The Human Epidermal Basement Membrane: A Shaped and Cell Instructive Platform That Aging Slowly Alters, Biomolecules. (2020) 10, no. 12, 10.3390/biom10121607, 1607. 33260936 PMC7760980

[25] Miskin R. P. and DiPersio C. M. , Roles for Epithelial Integrin *α*3*β*1 in Regulation of the Microenvironment During Normal and Pathological Tissue Remodeling, American Journal of Physiology-Cell Physiology. (2024) 326, no. 5, C1308–C1319, 10.1152/ajpcell.00128.2024. 38497112 PMC11371326

[26] Mercurio A. M. , Rabinovitz I. , and Shaw L. M. , The *α*6*β*4 Integrin and Epithelial Cell Migration, Current Opinion in Cell Biology. (2001) 13, no. 5, 541–545, 10.1016/S0955-0674(00)00249-0, 2-s2.0-0035479918. 11544021

[27] Kim K. K. , Wei Y. , and Szekeres C. , et al.Epithelial Cell *α*3*β*1 Integrin Links *β*-Catenin and Smad Signaling to Promote Myofibroblast Formation and Pulmonary Fibrosis, The Journal of Clinical Investigation. (2008) 119, no. 1, 213–224, 10.1172/jci36940, 2-s2.0-61749102618. 19104148 PMC2613463

[28] Xu M. , Tchkonia T. , and Kirkland J. L. , Perspective: Targeting the JAK/STAT Pathway to Fight Age-Related Dysfunction, Pharmacological Research. (2016) 111, 152–154, 10.1016/j.phrs.2016.05.015, 2-s2.0-84975840886. 27241018 PMC5026572

[29] Kalluri R. and Weinberg R. A. , The Basics of Epithelial-Mesenchymal Transition, Journal of Clinical Investigation. (2009) 119, no. 6, 1420–1428, 10.1172/JCI39104, 2-s2.0-67650999875. 19487818 PMC2689101

[30] Xin P. , Xu X. , and Deng C. , et al.The Role of JAK/STAT Signaling Pathway and Its Inhibitors in Diseases, International Immunopharmacology. (2020) 80, 10.1016/j.intimp.2020.106210, 106210. 31972425

[31] Mutlu A. S. , Duffy J. , and Wang M. C. , Lipid Metabolism and Lipid Signals in Aging and Longevity, Developmental Cell. (2021) 56, no. 10, 1394–1407, 10.1016/j.devcel.2021.03.034. 33891896 PMC8173711

[32] Ando Y. , Lazarus G. S. , and Jensen P. J. , Activation of Protein Kinase C Inhibits Human Keratinocyte Migration, Journal of Cellular Physiology. (1993) 156, no. 3, 487–496, 10.1002/jcp.1041560308, 2-s2.0-0027228129. 8360256

[33] Zhang X. , Yin M. , and Zhang L.-J. , Keratin 6, 16 and 17—Critical Barrier Alarmin Molecules in Skin Wounds and Psoriasis, Cells. (2019) 8, no. 8, 10.3390/cells8080807, 807. 31374826 PMC6721482

[34] Gu L.-H. and Coulombe P. A. , Keratin Function in Skin Epithelia: A Broadening Palette With Surprising Shades, Current Opinion in Cell Biology. (2007) 19, no. 1, 13–23, 10.1016/j.ceb.2006.12.007, 2-s2.0-33846307476. 17178453

[35] Rinnerthaler M. , Bischof J. , Streubel M. K. , Trost A. , and Richter K. , Oxidative Stress in Aging Human Skin, Biomolecules. (2015) 5, no. 2, 545–589, 10.3390/biom5020545, 2-s2.0-85012083478. 25906193 PMC4496685

[36] Oh J. , Lee Y. D. , and Wagers A. J. , Stem Cell Aging: Mechanisms, Regulators and Therapeutic Opportunities, Nature Medicine. (2014) 20, no. 8, 870–880, 10.1038/nm.3651, 2-s2.0-84905741423. PMC4160113 25100532

[37] Franceschi C. , Bonafè M. , and Valensin S. , et al.Inflamm-Aging: An Evolutionary Perspective on Immunosenescence, Annals of the New York Academy of Sciences. (2000) 908, no. 1, 244–254, 10.1111/j.1749-6632.2000.tb06651.x. 10911963

[38] Yeung Y. T. , Guerrero-Castilla A. , Cano M. , Muñoz M. F. , Ayala A. , and Argüelles S. , Dysregulation of the Hippo Pathway Signaling in Aging and Cancer, Pharmacological Research. (2019) 143, 151–165, 10.1016/j.phrs.2019.03.018, 2-s2.0-85063682070. 30910741

[39] Palazzo E. , Morandi P. , and Lotti R. , et al.Notch Cooperates With Survivin to Maintain Stemness and to Stimulate Proliferation in Human Keratinocytes During Ageing, International Journal of Molecular Sciences. (2015) 16, no. 11, 26291–26302, 10.3390/ijms161125948, 2-s2.0-84946616233. 26540052 PMC4661807

[40] Doles J. , Storer M. , Cozzuto L. , Roma G. , and Keyes W. M. , Age-Associated Inflammation Inhibits Epidermal Stem Cell Function, Genes & Development. (2012) 26, no. 19, 2144–2153, 10.1101/gad.192294.112, 2-s2.0-84867208183. 22972935 PMC3465736

[41] Dharan R. , Huang Y. , and Cheppali S. K. , et al.Tetraspanin 4 Stabilizes Membrane Swellings and Facilitates Their Maturation Into Migrasomes, Nature Communications. (2023) 14, no. 1, 10.1038/s41467-023-36596-9, 1037. PMC9950420 36823145

[42] Tu H. , Shi Y. , and Guo Y. , et al.Young Fibroblast-Derived Migrasomes Alleviate Keratinocyte Senescence and Enhance Wound Healing in Aged Skin, Journal of Nanobiotechnology. (2025) 23, no. 1, 10.1186/s12951-025-03293-2, 200. 40069826 PMC11895310

[43] Lee M.-J. , Byun M. R. , Furutani-Seiki M. , Hong J.-H. , and Jung H.-S. , YAP and TAZ Regulate Skin Wound Healing, Journal of Investigative Dermatology. (2014) 134, no. 2, 518–525, 10.1038/jid.2013.339, 2-s2.0-84892818938. 24108406

[44] Tayem R. , Niemann C. , and Pesch M. , et al.Laminin 332 Is Indispensable for Homeostatic Epidermal Differentiation Programs, Journal of Investigative Dermatology. (2021) 141, no. 11, 2602–2610.e3, 10.1016/j.jid.2021.04.008. 33965403

[45] Tzu J. and Marinkovich M. P. , Bridging Structure With Function: Structural, Regulatory, and Developmental Role of Laminins, The International Journal of Biochemistry & Cell Biology. (2008) 40, no. 2, 199–214, 10.1016/j.biocel.2007.07.015, 2-s2.0-36249022091. 17855154 PMC2192629

[46] Elbediwy A. , Vincent-Mistiaen Z. I. , and Spencer-Dene B. , et al.Integrin Signalling Regulates YAP and TAZ to Control Skin Homeostasis, Development. (2016) 143, no. 10, 1674–1687, 10.1242/dev.133728, 2-s2.0-84968861346. 26989177 PMC4874484

[47] Wang Y. , Yu A. , and Yu F.-X. , The Hippo Pathway in Tissue Homeostasis and Regeneration, Protein & Cell. (2017) 8, no. 5, 349–359, 10.1007/s13238-017-0371-0, 2-s2.0-85010756889. 28130761 PMC5413598

[48] Jones D. L. , Hallström G. F. , and Jiang X. , et al.Mechanoepigenetic Regulation of Extracellular Matrix Homeostasis via Yap and Taz, Proceedings of the National Academy of Sciences. (2023) 120, no. 22, 10.1073/pnas.2211947120, e2211947120. PMC10235980 37216538

[49] Pankratova M. D. , Riabinin A. A. , and Butova E. A. , et al.YAP/TAZ Signalling Controls Epidermal Keratinocyte Fate, International Journal of Molecular Sciences. (2024) 25, no. 23, 10.3390/ijms252312903, 12903. 39684613 PMC11641583

[50] De Rosa L. , Secone Seconetti A. , and De Santis G. , et al.Laminin 332-Dependent YAP Dysregulation Depletes Epidermal Stem Cells in Junctional Epidermolysis Bullosa, Cell Reports. (2019) 27, no. 7, 2036–2049.e6, 10.1016/j.celrep.2019.04.055, 2-s2.0-85065036273. 31091444

[51] Tang X. , Wang J. , and Chen J. , et al.Epidermal Stem Cells: Skin Surveillance and Clinical Perspective, Journal of Translational Medicine. (2024) 22, no. 1, 10.1186/s12967-024-05600-1, 779. 39169334 PMC11340167

[52] Takazawa Y. , Ogawa E. , and Saito R. , et al.Notch Down-Regulation in Regenerated Epidermis Contributes to Enhanced Expression of Interleukin-36*α* and Suppression of Keratinocyte Differentiation During Wound Healing, Journal of Dermatological Science. (2015) 79, no. 1, 10–19, 10.1016/j.jdermsci.2015.04.003, 2-s2.0-84929945511. 25982147

[53] Grönniger E. , Max H. , and Lyko F. , Skin Rejuvenation by Modulation of DNA Methylation, Experimental Dermatology. (2024) 33, no. 10, 10.1111/exd.70005, e70005. 39440959

[54] Haykal D. , Flament F. , Mora P. , Balooch G. , and Cartier H. , Unlocking Longevity in Aesthetic Dermatology: Epigenetics, Aging, and Personalized Care, International Journal of Dermatology. (2025) 64, no. 12, 2204–2214, 10.1111/ijd.17725. 40064617 PMC12605702

[55] Lin C. , Li H. , and Liu J. , et al.Arginine Hypomethylation-Mediated Proteasomal Degradation of Histone H4—An Early Biomarker of Cellular Senescence, Cell Death and Differentiation. (2020) 27, no. 9, 2697–2709, 10.1038/s41418-020-0562-8. 32447347 PMC7429905

[56] De Cecco M. , Criscione S. W. , and Peckham E. J. , et al.Genomes of Replicatively Senescent Cells Undergo Global Epigenetic Changes Leading to Gene Silencing and Activation of Transposable Elements, Aging Cell. (2013) 12, no. 2, 247–256, 10.1111/acel.12047, 2-s2.0-84878954836. 23360310 PMC3618682

[57] Cruickshanks H. A. , McBryan T. , and Nelson D. M. , et al.Senescent Cells Harbour Features of the Cancer Epigenome, Nature Cell Biology. (2013) 15, no. 12, 1495–1506, 10.1038/ncb2879, 2-s2.0-84893397675. 24270890 PMC4106249

[58] Lyko F. , The DNA Methyltransferase Family: A Versatile Toolkit for Epigenetic Regulation, Nature Reviews Genetics. (2018) 19, no. 2, 81–92, 10.1038/nrg.2017.80, 2-s2.0-85040772858. 29033456

[59] Jurkowska R. Z. and Jeltsch A. , Enzymology of Mammalian DNA Methyltransferases, Advances in Experimental Medicine and Biology. (2022) 1389, 69–110, 10.1007/978-3-031-11454-0_4. 36350507

[60] Falckenhayn C. , Bienkowska A. , and Söhle J. , et al.Identification of Dihydromyricetin as a Natural DNA Methylation Inhibitor With Rejuvenating Activity in Human Skin, Frontiers in Aging. (2024) 4, 10.3389/fragi.2023.1258184, 1258184. 38500495 PMC10944877

[61] Qian Y. , Tu J. , and Tang N. L. S. , et al.Dynamic Changes of DNA Epigenetic Marks in Mouse Oocytes During Natural and Accelerated Aging, The International Journal of Biochemistry & Cell Biology. (2015) 67, 121–127, 10.1016/j.biocel.2015.05.005, 2-s2.0-84940889663. 25982203

[62] Yang E. , Juan Z. , and Hengshu Z. , Mechanism of SPRY1 Methylation Regulating Natural Aging of Skin Epidermal Cells, Journal of Cosmetic Dermatology. (2020) 19, no. 5, 1224–1230, 10.1111/jocd.13126, 2-s2.0-85071753656. 31483543

[63] Beck D. B. , Petracovici A. , and He C. , et al.Delineation of a Human Mendelian Disorder of the DNA Demethylation Machinery: TET3 Deficiency, The American Journal of Human Genetics. (2020) 106, no. 2, 234–245, 10.1016/j.ajhg.2019.12.007. 31928709 PMC7010978

[64] Qiu B. , Yang E. , Zheng Y. , and Zhang H. , Association Between SPRY1 and TET3 in Skin Photoaging and Natural Aging Mechanisms, Journal of Cosmetic Dermatology. (2024) 23, no. 4, 1396–1403, 10.1111/jocd.16115. 38054565

[65] Flament F. , Maudet A. , and Bayer-Vanmoen M. , The Objective and Subjective Impact of a Daily Self-Massage on Visible Signs of Stress on the Skin and Emotional Well-Being, International Journal of Cosmetic Science. (2023) 45, no. 6, 761–768, 10.1111/ics.12884. 37483121

[66] Kim H.-Y. , Lee D. H. , Shin M. H. , Shin H. S. , Kim M.-K. , and Chung J. H. , UV-Induced DNA Methyltransferase 1 Promotes Hypermethylation of Tissue Inhibitor of Metalloproteinase 2 in the Human Skin, Journal of Dermatological Science. (2018) 91, no. 1, 19–27, 10.1016/j.jdermsci.2018.03.009, 2-s2.0-85045938792. 29685765

[67] Wang B. , Han J. , Elisseeff J. H. , and Demaria M. , The Senescence-Associated Secretory Phenotype and Its Physiological and Pathological Implications, Nature Reviews Molecular Cell Biology. (2024) 25, no. 12, 958–978, 10.1038/s41580-024-00727-x. 38654098

[68] Suryadevara V. , Hudgins A. D. , and Rajesh A. , et al.SenNet Recommendations for Detecting Senescent Cells in Different Tissues, Nature Reviews Molecular Cell Biology. (2024) 25, no. 12, 1001–1023, 10.1038/s41580-024-00738-8. 38831121 PMC11578798

[69] Fiorentino F. P. , Tokgün E. , and Solé-Sánchez S. , et al.Growth Suppression by MYC Inhibition in Small Cell Lung Cancer Cells With TP53 and RB1 Inactivation, Oncotarget. (2016) 7, no. 21, 31014–31028, 10.18632/oncotarget.8826, 2-s2.0-84971508916. 27105536 PMC5058735

[70] Hu W. , Jing Y. , Yu Q. , and Huang N. , Differential Gene Screening and Bioinformatics Analysis of Epidermal Stem Cells and Dermal Fibroblasts During Skin Aging, Scientific Reports. (2022) 12, no. 1, 10.1038/s41598-022-16314-z, 12019. 35835980 PMC9283434

[71] Ogata Y. , Yamada T. , and Hasegawa S. , et al.SASP-Induced Macrophage Dysfunction May Contribute to Accelerated Senescent Fibroblast Accumulation in the Dermis, Experimental Dermatology. (2021) 30, no. 1, 84–91, 10.1111/exd.14205. 33010063

[72] Cordisco S. , Maurelli R. , and Bondanza S. , et al.Bmi-1 Reduction Plays a Key Role in Physiological and Premature Aging of Primary Human Keratinocytes, Journal of Investigative Dermatology. (2010) 130, no. 4, 1048–1062, 10.1038/jid.2009.355, 2-s2.0-77949539975. 19907431

[73] Ressler S. , Bartkova J. , and Niederegger H. , et al.p16^INK4A^ Is a Robust In Vivo Biomarker of Cellular Aging in Human Skin, Aging Cell. (2006) 5, no. 5, 379–389, 10.1111/j.1474-9726.2006.00231.x, 2-s2.0-33748327046. 16911562

[74] Wang A. S. , Ong P. F. , Chojnowski A. , Clavel C. , and Dreesen O. , Loss of Lamin B1 Is a Biomarker to Quantify Cellular Senescence in Photoaged Skin, Scientific Reports. (2017) 7, no. 1, 10.1038/s41598-017-15901-9, 2-s2.0-85034435023, 15678. 29142250 PMC5688158

[75] Rouillard M. E. , Hu J. , Sutter P. A. , Kim H. W. , Huang J. K. , and Crocker S. J. , The Cellular Senescence Factor Extracellular HMGB1 Directly Inhibits Oligodendrocyte Progenitor Cell Differentiation and Impairs CNS Remyelination, Frontiers in Cellular Neuroscience. (2022) 16, 10.3389/fncel.2022.833186, 833186. 35573828 PMC9095917

[76] Jing Y. , Jiang X. , and Ji Q. , et al.Genome-Wide CRISPR Activation Screening in Senescent Cells Reveals SOX5 as a Driver and Therapeutic Target of Rejuvenation, Cell Stem Cell. (2023) 30, no. 11, 1452–1471.e10, 10.1016/j.stem.2023.09.007. 37832549

[77] Kim H. J. , Jin S. P. , and Kang J. , et al.Uncovering the Impact of UV Radiation on Mitochondria in Dermal Cells: A STED Nanoscopy Study, Scientific Reports. (2024) 14, no. 1, 10.1038/s41598-024-55778-z, 8675. 38622160 PMC11018800

[78] Valsecchi F. , Koopman W. J. H. , Manjeri G. R. , Rodenburg R. J. , Smeitink J. A. M. , and Willems P. H. G. M. , Complex I Disorders: Causes, Mechanisms, and Development of Treatment Strategies at the Cellular Level, Developmental Disabilities Research Reviews. (2010) 16, no. 2, 175–182, 10.1002/ddrr.107, 2-s2.0-77956232845. 20818732

[79] Koopman W. J. H. , Visch H.-J. , Verkaart S. , van den Heuvel L. W. P. J. , Smeitink J. A. M. , and Willems P. H. G. M. , Mitochondrial Network Complexity and Pathological Decrease in Complex I Activity Are Tightly Correlated in Isolated Human Complex I Deficiency, American Journal of Physiology-Cell Physiology. (2005) 289, no. 4, C881–C890, 10.1152/ajpcell.00104.2005, 2-s2.0-25444446126. 15901599

[80] Valsecchi F. , Monge C. , and Forkink M. , et al.Metabolic Consequences of NDUFS4 Gene Deletion in Immortalized Mouse Embryonic Fibroblasts, Biochimica et Biophysica Acta (BBA) - Bioenergetics. (2012) 1817, no. 10, 1925–1936, 10.1016/j.bbabio.2012.03.006, 2-s2.0-84864701822. 22430089

[81] Spehar K. , Pan A. , and Beerman I. , Restoring Aged Stem Cell Functionality: Current Progress and Future Directions, Stem Cells. (2020) 38, no. 9, 1060–1077, 10.1002/stem.3234. 32473067 PMC7483369

[82] Malaquin N. , Martinez A. , and Rodier F. , Keeping the Senescence Secretome Under Control: Molecular Reins on the Senescence-Associated Secretory Phenotype, Experimental Gerontology. (2016) 82, 39–49, 10.1016/j.exger.2016.05.010, 2-s2.0-84979633299. 27235851

[83] Jia Y. , Chen X. , and Sun J. , Apremilast Ameliorates IL-1*α*-Induced Dysfunction in Epidermal Stem Cells, Aging. (2021) 13, no. 15, 19293–19305, 10.18632/aging.203265. 34375302 PMC8386542

[84] Liu Y. , Ho C. , and Wen D. , et al.Targeting the Stem Cell Niche: Role of Collagen XVII in Skin Aging and Wound Repair, Theranostics. (2022) 12, no. 15, 6446–6454, 10.7150/thno.78016. 36185608 PMC9516244

[85] Xiang Y. , Liu Y. , and Yang Y. , et al.Reduced Expression of Collagen 17A1 in Naturally Aged, Photoaged, and UV-Irradiated Human Skin In Vivo: Potential Links to Epidermal Aging, Journal of Cell Communication and Signaling. (2022) 16, no. 3, 421–432, 10.1007/s12079-021-00654-y. 35060094 PMC9411357

[86] Xu L.-W. , Sun Y.-D. , and Fu Q.-Y. , et al.Unveiling Senescence-Associated Secretory Phenotype in Epidermal Aging: Insights From Reversibly Immortalized Keratinocytes, Aging. (2024) 16, no. 18, 12651–12666, 10.18632/aging.206117. 39316420 PMC11466489

[87] Coppé J.-P. , Desprez P.-Y. , Krtolica A. , and Campisi J. , The Senescence-Associated Secretory Phenotype: The Dark Side of Tumor Suppression, Annual Review of Pathology: Mechanisms of Disease. (2010) 5, no. 1, 99–118, 10.1146/annurev-pathol-121808-102144, 2-s2.0-77949881221. PMC4166495 20078217

[88] Watanabe M. , Kosumi H. , and Osada S. I. , et al.Type XVII Collagen Interacts With the aPKC-PAR Complex and Maintains Epidermal Cell Polarity, Experimental Dermatology. (2021) 30, no. 1, 62–67, 10.1111/exd.14196. 32970880

[89] Ryan C. P. , Epigenetic Clocks”: Theory and Applications in Human Biology, American Journal of Human Biology. (2021) 33, no. 3, 10.1002/ajhb.23488, e23488. 32845048

[90] Lee H. Y. , Lee S. D. , and Shin K.-J. , Forensic DNA Methylation Profiling From Evidence Material for Investigative Leads, BMB Reports. (2016) 49, no. 7, 359–369, 10.5483/BMBRep.2016.49.7.070, 2-s2.0-84991111273. 27099236 PMC5032003

[91] Jylhävä J. , Pedersen N. L. , and Hägg S. , Biological Age Predictors, EBioMedicine. (2017) 21, 29–36, 10.1016/j.ebiom.2017.03.046, 2-s2.0-85017131847. 28396265 PMC5514388

[92] Declerck K. and Vanden Berghe W. , Back to the Future: Epigenetic Clock Plasticity Towards Healthy Aging, Mechanisms of Ageing and Development. (2018) 174, 18–29, 10.1016/j.mad.2018.01.002, 2-s2.0-85040961623. 29337038

[93] Horvath S. , Oshima J. , and Martin G. M. , et al.Epigenetic Clock for Skin and Blood Cells Applied to Hutchinson Gilford Progeria Syndrome and Ex Vivo Studies, Aging. (2018) 10, no. 7, 1758–1775, 10.18632/aging.101508, 2-s2.0-85050992380. 30048243 PMC6075434

[94] Roig-Genoves J. V. , García-Giménez J. L. , and Mena-Molla S. , A miRNA-Based Epigenetic Molecular Clock for Biological Skin-Age Prediction, Archives of Dermatological Research. (2024) 316, no. 6, 10.1007/s00403-024-03129-3, 326. 38822910 PMC11144124

[95] Xie Q. , Bai Q. , and Zou L.-Y. , et al.Genistein Inhibits DNA Methylation and Increases Expression of Tumor Suppressor Genes in Human Breast Cancer Cells, Genes, Chromosomes and Cancer. (2014) 53, no. 5, 422–431, 10.1002/gcc.22154, 2-s2.0-84895922467. 24532317

[96] Ming T. , Tao Q. , and Tang S. , et al.An Epigenetic Regulator and Its Application in Cancer, Biomedicine & Pharmacotherapy. (2022) 156, 10.1016/j.biopha.2022.113956, 113956. 36411666

[97] Qaria M. A. , Xu C. , and Hu R. , et al.Ectoine Globally Hypomethylates DNA in Skin Cells and Suppresses Cancer Proliferation, Marine Drugs. (2023) 21, no. 12, 10.3390/md21120621, 621. 38132942 PMC10744768

[98] Albouy M. , Aubailly S. , and Jeanneton O. , et al.Skin-Protective Biological Activities of Bio-Fermented *Aframomum angustifolium* Extract by a Consortium of Microorganisms, Frontiers in Pharmacology. (2023) 14, 10.3389/fphar.2023.1303198, 1303198. 38186646 PMC10768170

[99] Dong Y. , Zhang Y. , and Yu H. , et al.Poly-l-Lactic Acid Microspheres Delay Aging of Epidermal Stem Cells in Rat Skin, Frontiers in Immunology. (2024) 15, 10.3389/fimmu.2024.1394530, 1394530. 38881903 PMC11177849

[100] Alcaraz A. , Mrowiec A. , and Insausti C. L. , et al.Amniotic Membrane Modifies the Genetic Program Induced by TGFß, Stimulating Keratinocyte Proliferation and Migration in Chronic Wounds, PLoS One. (2015) 10, no. 8, 10.1371/journal.pone.0135324, 2-s2.0-84942645678, e0135324. 26284363 PMC4540284

[101] Ghatak S. , Chan Y. C. , and Khanna S. , et al.Barrier Function of the Repaired Skin Is Disrupted Following Arrest of Dicer in Keratinocytes, Molecular Therapy. (2015) 23, no. 7, 1201–1210, 10.1038/mt.2015.65, 2-s2.0-84934435564. 25896246 PMC4817784

[102] Vidali S. , Chéret J. , and Giesen M. , et al.Thyroid Hormones Enhance Mitochondrial Function in Human Epidermis, Journal of Investigative Dermatology. (2016) 136, no. 10, 2003–2012, 10.1016/j.jid.2016.05.118, 2-s2.0-84996969416. 27349864

